# Bioactive hemostatic materials: a new strategy for promoting wound healing and tissue regeneration

**DOI:** 10.1002/mco2.70113

**Published:** 2025-03-22

**Authors:** Zhengyuan Liu, Junnan Xu, Xing Wang

**Affiliations:** ^1^ Beijing National Laboratory for Molecular Sciences Institute of Chemistry Chinese Academy of Sciences Beijing China; ^2^ Sino‐Danish College University of Chinese Academy of Sciences (UCAS) Beijing China; ^3^ Nano‐Science Center University of Copenhagen Copenhagen Denmark; ^4^ Department of Urology the Third Medical Center of PLA General Hospital Beijing China

**Keywords:** biomaterials, multifunctional wound healing materials, smart dressings, wound healing

## Abstract

Wound healing remains a critical global healthcare challenge, with an annual treatment cost exceeding $50 billion worldwide. Over the past decade, significant advances in wound care have focused on developing sophisticated biomaterials that promote tissue regeneration and prevent complications. Despite these developments, there remains a crucial need for multifunctional wound healing materials that can effectively address the complex, multiphase nature of wound repair while being cost effective and easily applicable in various clinical settings. This review systematically analyzes the latest developments in wound healing materials, examining their chemical composition, structural design, and therapeutic mechanisms. We comprehensively evaluate various bioactive components, including natural polymers, synthetic matrices, and hybrid composites, along with their different forms, such as hydrogels, powders, and smart dressings. Special attention is given to emerging strategies in material design that integrate multiple therapeutic functions, including sustained drug delivery, infection prevention, and tissue regeneration promotion. The insights provided in this review illuminate the path toward next‐generation wound healing materials, highlighting opportunities for developing more effective therapeutic solutions that can significantly improve patient outcomes and reduce healthcare burden.

## Graphical abstract

This paper reviews the current status and latest advancements in the field of hemostatic materials, focusing on the mechanism of hemostasis and wound healing, the chemical design principles and physiological mechanisms of hemostatic materials, and the comparison of different active ingredients and dosage forms. In addition, considering the subsequent needs of wound healing posthemostasis, this review also discusses the main challenges and future directions of hemostatic materials for wound healing

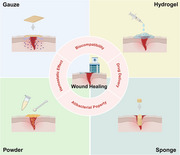

## 1 INTRODUCTION

Wound healing has been a critical focus in medical care throughout history, evolving from simple bandages to sophisticated bioactive materials.^1^ As a fundamental physiological process, wound healing not only aids in tissue recovery but also provides essential protection against pathogenic invasion.^2^, ^3^ The global wound care market has experienced substantial growth, reaching $20.5 billion in 2023, driven by an aging population, increasing prevalence of chronic wounds, and rising surgical procedures.^4^, ^5^ This significant healthcare burden has stimulated intensive research into advanced wound healing technologies and materials.

The development of wound healing materials has progressed through several distinct phases, from traditional passive dressings to current smart, multifunctional materials. Hemostasis, as the initial and crucial stage of wound healing, requires materials with specific characteristics: excellent biocompatibility, rapid hemostatic action, and strong tissue adhesion under wet conditions.^1^, ^6^, ^7^ Despite significant advances, current commercial products still face challenges, including inconsistent performance, poor biocompatibility, and high costs. Recent research has focused on developing advanced materials incorporating various active compounds, such as natural polymers (e.g., chitosan [CS]), synthetic polymers, inorganic materials, and metal‐containing composites.^8^, ^9^ These materials demonstrate diverse mechanisms of action, from promoting platelet activation to enhancing fibrin formation, while some also provide additional benefits such as antimicrobial properties.^10^, ^11^

Given the rapid evolution of wound healing materials and their expanding therapeutic applications, there is a pressing need to analyze recent developments and emerging trends in this field systematically. This review aims to provide comprehensive insights into the current state of wound healing materials, with particular emphasis on innovative design strategies and their clinical implications. We focus on examining how different material compositions and structures influence healing outcomes and how these insights can guide future development.

The review is structured to present a logical progression through key aspects of wound healing materials. We begin by examining the fundamental mechanisms of wound healing and hemostasis, providing essential context for material design considerations. This is followed by a detailed analysis of current clinical and military applications, including both conventional and advanced products. We then explore innovative design strategies and expanded functions of various material forms, particularly focusing on their therapeutic mechanisms and performance optimization. The review concludes with a critical discussion of current challenges and future prospects, highlighting promising directions for research and development that could lead to more effective wound healing solutions.

## 2 HEMOSTASIS AND WOUND HEALING MECHANISMS

### 2.1 Four stages of wound healing

**FIGURE 1:**
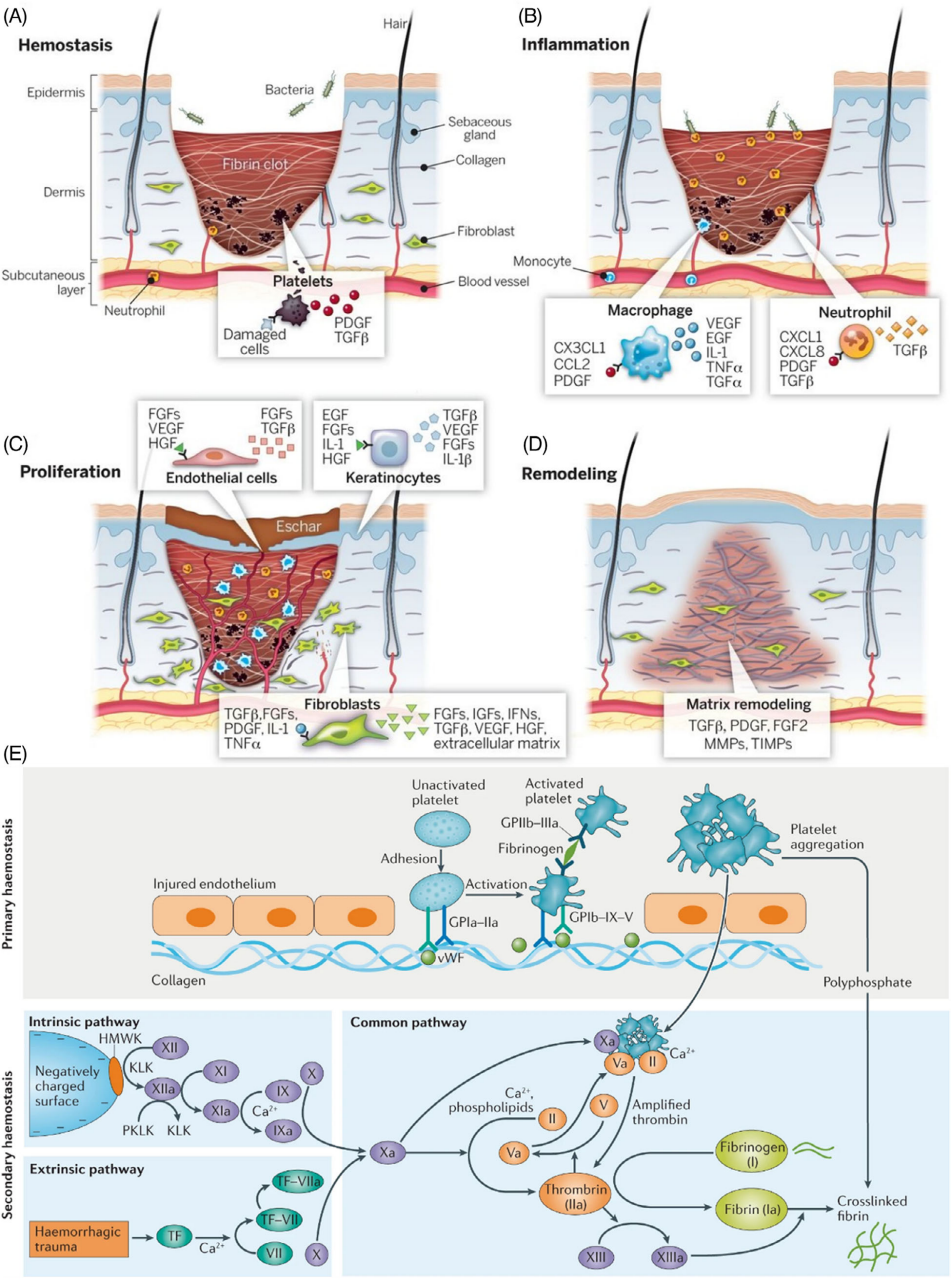
Four stages in wound healing. (A) Hemostasis, (B) inflammation, (C) proliferation, and (D) remodeling.^19^ Reproduced with permission, © The American Association for the Advancement of Science. (E) Mechanisms of primary and secondary hemostasis.^1^ Reproduced with permission, © Springer Nature Limited.

The wound healing process represents an intricate biological cascade that progresses through four interconnected stages (hemostasis, inflammation, proliferation, and remodeling; Figure 1),^12^ each characterized by distinct cellular and molecular events. The initial hemostasis stage begins immediately upon injury, where platelets rapidly aggregate at the wound site to stimulate the form of a fibrin clot.^13^ This provisional matrix not only prevents excessive blood loss but also serves as a scaffold for incoming cells while releasing crucial growth factors such as PDGF and TGFβ from damaged cells.^14^, ^15^ Neutrophils are simultaneously recruited to the wound site, initiating the inflammatory response.^16^, ^17^, ^18^

The inflammatory stage is marked by the orchestrated invasion of immune cells, primarily macrophages and neutrophils, which coordinate the release of a complex array of inflammatory mediators and growth factors.^20^ Macrophages secrete chemokine C‐X3‐C motif ligand 1 (CX3CL1), C‐C motif chemokine ligand 2 (CCL2), platelet‐derived growth factor (PDGF), vascular endothelial growth factor (VEGF), epidermal growth factor (EGF), interleukin‐1 (IL‐1), tumor necrosis factor alpha (TNFα), and transforming growth factor alpha (TGFα),^21^, ^22^, ^23^ while neutrophils release while neutrophils release C‐X‐C motif chemokine ligand 1 (CXCL1), C‐X‐C motif chemokine ligand 8 (CXCL8), PDGF, and transforming growth factor beta (TGFβ).^23^, ^24^, ^25^ These factors collectively regulate the inflammatory response for promoting the wound environment for tissue regeneration.

During the proliferation stage, the wound undergoes significant cellular activity and tissue formation.^26^, ^27^ Endothelial cells become activated and gradually release the FGFs, VEGF, and HGF to promote angiogenesis. Simultaneously, keratinocytes produce EGF, FGFs, IL‐1, and HGF to facilitate reepithelialization, while fibroblasts secrete TGFβ, FGFs, PDGF, IL‐1, and TNFα to support new extracellular matrix (ECM) formation.^28^, ^29^, ^30^, ^31^ This coordinates the cellular response results in the formation of granulation tissue, providing the foundation for wound closure.

The final remodeling stage involves the systematic reorganization of the wound matrix, primarily regulated by TGFβ, PDGF, and FGF2.^32^ This stage is characterized by the balanced activity of MMPs and TIMPs, leading to collagen remodeling and wound contraction, and ultimately resulting in scar tissue formation.^27^

### 2.2 Primary and secondary hemostasis

Hemostasis represents a critical physiological response to vascular injury, orchestrating both immediate blood loss control and subsequent wound healing through two interconnected stages: primary and secondary hemostasis (Figure 1E).^33^ In primary hemostasis, the exposure of subendothelial collagen following vascular injury triggers an immediate platelet response. This process begins with platelet adhesion to the damaged endothelium through specific membrane receptors: GPIa‐IIa directly binds to exposed collagen, while von Willebrand factor mediates additional platelet recruitment via GPIb‐IX‐V receptors under high shear conditions.^34^, ^35^, ^36^ Upon adhesion, platelets undergo the activation, leading to conformational changes in GPIIb‐IIIa receptors that enable fibrinogen binding and subsequent platelet aggregation, ultimately forming an initial platelet plug.^37^

Secondary hemostasis reinforces this initial response through a complex cascade of coagulation factors proceeding via two distinct yet converging pathways.^38^ The intrinsic pathway initiates when blood contacts negatively charged surfaces, triggering a sequential activation cascade beginning with high‐molecular‐weight kininogen and kallikrein, proceeding through factors XII, XI, and IX.^39^, ^40^ Concurrently, the extrinsic pathway activates in response to tissue injury, where tissue factor complexes with factor VII in the presence of Ca^2+^ ions. Both pathways converge at factor X activation, forming the common pathway where factor Xa combines with factor Va to form the prothrombinase complex. This complex catalyzes the conversion of prothrombin to thrombin, which then cleaves fibrinogen to fibrin monomers.^41^, ^42^, ^43^ Finally, factor XIII catalyzes the cross‐linking of these fibrin monomers, creating a stable fibrin mesh that reinforces the initial platelet plug.^37^, ^43^ The coordinated action of these pathways, facilitated by various cellular and molecular components, ensures rapid and effective hemostasis while providing a provisional matrix for subsequent wound healing processes.

## 3 POLYMERS FOR WOUND HEALING

Polymers represent a versatile class of biomaterials with tunable physicochemical properties and structural diversity that play crucial roles in modern wound care management. These materials can be engineered to mimic the natural ECM and promote tissue regeneration through various healing mechanisms. Their ability to absorb wound exudates, maintain moisture balance, and protect wounds from external contamination makes them ideal candidates for wound dressing applications.^44^, ^45^

The fabrication of polymer‐based wound dressings typically involves two main approaches: physical and chemical cross‐linking. Physical cross‐linking utilizes noncovalent interactions such as hydrogen bonding, ionic interactions, and chain entanglements to create functional composites.^46^, ^47^ Chemical modification methods include grafting, crosslinking, and surface functionalization, which can introduce specific bioactive groups or adjust material properties.^47^, ^48^ The selection of appropriate fabrication strategies is crucial for achieving desired wound healing outcomes.

Polymer sources can be broadly classified into natural and synthetic origins. Natural polymers like collagen, CS, and alginate exhibit inherent biocompatibility and cell recognition properties but often suffer from batch‐to‐batch variability, limited mechanical strength, and processing challenges.^49^ Synthetic polymers such as polyethylene glycol (PEG), poly(vinyl alcohol) (PVA), and polyurethanes offer consistent quality, controllable properties, and scalable production but may lack biological cues and biodegradability.^50^, ^51^ This has driven researchers to develop hybrid systems that combine the advantages of both natural and synthetic polymers while minimizing their respective limitations.

### 3.1 Natural polymer hydrogels

Natural polymers, originating from plants, animals, or microbes, are frequently used in biomedical applications because of their high biocompatibility and biological functions.^52^ As shown in Table 1, natural polymers possess inherent characteristics, including hydrophilicity, biocompatibility, and biodegradability. Hydrogels synthesized from natural polymers are comparable to ECMs.^53^

**TABLE 1:** Polymers for wound healing.

Type	Polymers	Synthesis method	Properties	Functional groups	References
Natural polymers	Cellulose	Trees, fruits, vegetables	Biocompatibility, biodegradability, renewability	Hydroxyl	^54^, ^55^, ^56^
	Chitosan	Crab shells, shrimp shells	Abundant, strong adsorption capacity, easy modification	Amino, hydroxyl	^57^, ^58^, ^59^
	Alginate	Brown algae, bacteria	Biocompatibility, plasticity	Carboxyl, hydroxyl	^60^, ^61^, ^62^
	Hyaluronic acid	Animal tissues, microbial fermentation	Biocompatibility, biodegradability, nontoxicity, nonimmunogenicity	Hydroxyl, carboxyl	^63^, ^64^, ^65^
	Collagen	Connective tissues of livestock and poultry	Biocompatibility, biodegradability, cell adhesion	Carboxyl, amino	^66^, ^67^
	Silk fibroin	Silkworm silk	Easy to purify, sterilize, process; biocompatibility, low immunogenicity, drug loading capacity	Carboxyl, amino, hydroxyl	^68^, ^69^, ^70^
	Dextran	Yeast, fungi, bacteria, grains	Biocompatibility, ability to reduce nonspecific protein adsorption and cell attachment	Vicinal hydroxyl	^71^, ^72^
Synthetic polymers	Polyethylene glycol (PEG)	Gradual addition reaction of ethylene oxide with water or ethylene glycol	Biocompatible, low toxicity, anticell protein adhesion	Hydroxyl	^61^, ^73^
	Polyacrylic acid (PAA)	Free radical polymerization of acrylic acid monomers in water	Water‐soluble, nontoxic, biocompatible	Carboxyl	^74^, ^75^, ^76^
	Polyvinyl alcohol (PVA)	Alcoholysis or hydrolysis of polyvinyl esters	Water‐soluble, biocompatible	Hydroxyl, acetate	^77^, ^78^, ^79^
	Polyacrylamide (PAM)	Polymerization of acrylamide monomers	Nontoxic, high toughness, easy to form hydrogen bonds	Amide	^80^, ^81^
	Block copolymer	Connection of hydrophilic and hydrophobic polymer segments	Stimuli‐responsive, self‐assembling, biocompatible	Determined by the block copolymer segments	^82^, ^83^
	Polypeptide	Ring‐opening polymerization of amino acids with N‐carboxyanhydrides	Biocompatible, structurally adjustable, degradable	Amino, hydroxyl, carboxyl	^84^, ^85^

### 3.2 Synthetic polymer hydrogels

Compared with natural polymers, synthetic polymers exhibit notable biofunctional differences. Synthetic polymers display more bioinertness and less biofunctionality (Table 1). Nevertheless, the mechanical strength of synthetic polymers is a significant advantage. It has been demonstrated that the mechanical properties of synthetic polymers can be further manipulated through cross‐linking or compounding with natural polymers.^86^, ^87^ Furthermore, the introduction of targeted functional groups for specific cross‐linking mechanisms and functions can be facilitated by the increased chemical reactivity of synthetic polymers.^88^, ^89^, ^90^ This property offers a broader option for the development of hydrogels.

## 4 PRODUCTS FOR CLINICAL AND MILITARY APPLICATIONS

Wound healing is a critical component in the treatment of trauma, surgery, and chronic wounds. Following an extensive study of wound healing mechanisms, various wound healing products for clinical applications have been developed. These products can be divided into different groups based on their origin, mechanism of action, and mode of application, ranging from conventional wound dressings to advanced bioactive materials (Table 2). These wound healing materials provide a wide range of options for clinical wound management by targeting different phases of the wound healing process. To optimize therapeutic outcomes, clinicians can choose the ideal wound healing product by considering the wound type, anatomical location, and healing stage. Notably, certain advanced wound healing products have been specifically designed for emergency medical services and disaster response scenarios, adhering to more rigorous standards to meet unique requirements, including efficacy, safety profile, ease of use, and durability.^91^ These specialized materials are engineered to maintain stability and portability during storage while delivering effective therapeutic outcomes in challenging environments.

**TABLE 2:** Clinic products: categories, examples, and descriptions.

Categories		Product examples	Description	References
Local agents	Fibrin adhesives	Tisseel, Evicel	The main components are fibrinogen and factor XIII, which form stable fibrin clots at the wound site to promote hemostasis.	^92^, ^93^
	Gelatin‐based sponges	Gelfoam, Surgifoam	It is made from gelatin with good biocompatibility and absorbability, which can fill wounds and provide physical mechanical hemostasis.	^94^, ^95^
	Oxidized cellulose products	Surgicel, Interceed	It has both hemostatic and antiadhesion effects and can be used for oozing hemorrhage and capillary bleeding.	^96^, ^97^
	Chitosan products	HemCon, Celox	It utilizes chitosan's positive charge to interact with red blood cells and platelets in the blood, promoting blood coagulation.	^91^, ^98^
	Prothrombin complex concentrates	Prothromplex, PPSB	It contains coagulation factors II, VII, IX, X, and so on, and can quickly stop bleeding caused by coagulation factor deficiencies.	^99^, ^100^
Blood‐derived agents	Platelet preparations	Platelet suspension, platelet concentrate	The number of platelets can increase platelet count and function, which is useful for bleeding caused by platelet decrease or dysfunction.	^56^, ^101^
Antifibrinolytic agents	Aminocaproic acid	Amicar	It can inhibit plasminogen activators and prevent fibrin dissolution. It is commonly used for abnormal bleeding caused by cardiac surgery, gynecological surgery, and so on.	^102^
	Tranexamic acid	Transamin	The mechanism of action is similar to aminocaproic acid, with wide clinical application.	^103^
Other agents	Vitamin K preparations	Menadione, ferrous fumarate	It can promote liver synthesis of coagulation factors, used for the prevention and treatment of vitamin K deficiency bleeding.	^104^, ^105^
	Thrombin	Reptilase	It directly converts fibrinogen to fibrin and can be used for surgical oozing and capillary bleeding.	^106^

By evaluating the current landscape of wound healing products, it becomes apparent that many conventional products exhibit relatively slow healing rates and limited efficacy, potentially failing to address the demands of complex wounds and chronic ulcers adequately. Furthermore, the usability of certain products is suboptimal, necessitating operation by trained medical professionals. Additionally, the use of these products, particularly those containing growth factors or biological components, carries inherent risks of immunogenicity and potential complications.^107^ While advanced wound healing products boast superior protective properties, their relatively high cost and specialized distribution channels often limit their accessibility in routine clinical care. Moreover, many current wound healing materials often overlook the integration of multiple therapeutic functions, such as infection prevention and tissue regeneration promotion. Consequently, the future development of wound healing products must strike a delicate balance between healing efficacy, safety, and ease of use while carefully considering and addressing the comprehensive aspects of wound care to ensure optimal patient outcomes.^108^

## 5 PROPERTIES OF WOUND HEALING MATERIALS

The significance of wound healing materials in clinical applications is of the utmost importance, particularly in surgery and emergency care, where rapid and effective bleeding control and the promotion of wound healing are crucial. To fulfill these requirements, wound healing materials must contain several essential properties.

### 5.1 Excellent biocompatibility

The biocompatibility of materials is closely associated with the safety of patients and the efficiency of clinical therapy.^109^ Biocompatible materials can reduce inflammatory reactions, immune rejection, toxicity, sensitization, and other negative responses. Additionally, they can stimulate tissue regeneration and repair and accelerate wound healing.^1^, ^3^ Peptide‐based self‐assembled hydrogels are biocompatible materials that use specific peptide sequences to form three‐dimensional network structures through self‐assembly.^110^, ^111^, ^112^ These structures imitate the ECM and offer the ideal cell growth and tissue regeneration environment.^113^ Bai et al. created a hydrogel by grafting dopamine onto poly(l‐glutamic acid) with enhanced bioactivity of the hydrogel and promoted cell adhesion and proliferation, and its antioxidant properties can reduce the effects of oxidative stress on wounds. Besides, peptide‐based material has been demonstrated to resist heat, salt, and urea.It can be easily separated from tissues with quick PBS washing, which makes it beneficial for clinical procedures and minimizes pain and secondary wound injury in patients.^115^ The safety and efficacy of materials depend on the biocompatibility and its degradation products. Clinical application and translation can be promoted only after an in‐depth evaluation of the biocompatibility of hemostatic materials' degradation products and ensuring their safety and efficacy.

### 5.2 Excellent hemostatic effect

**FIGURE 2:**
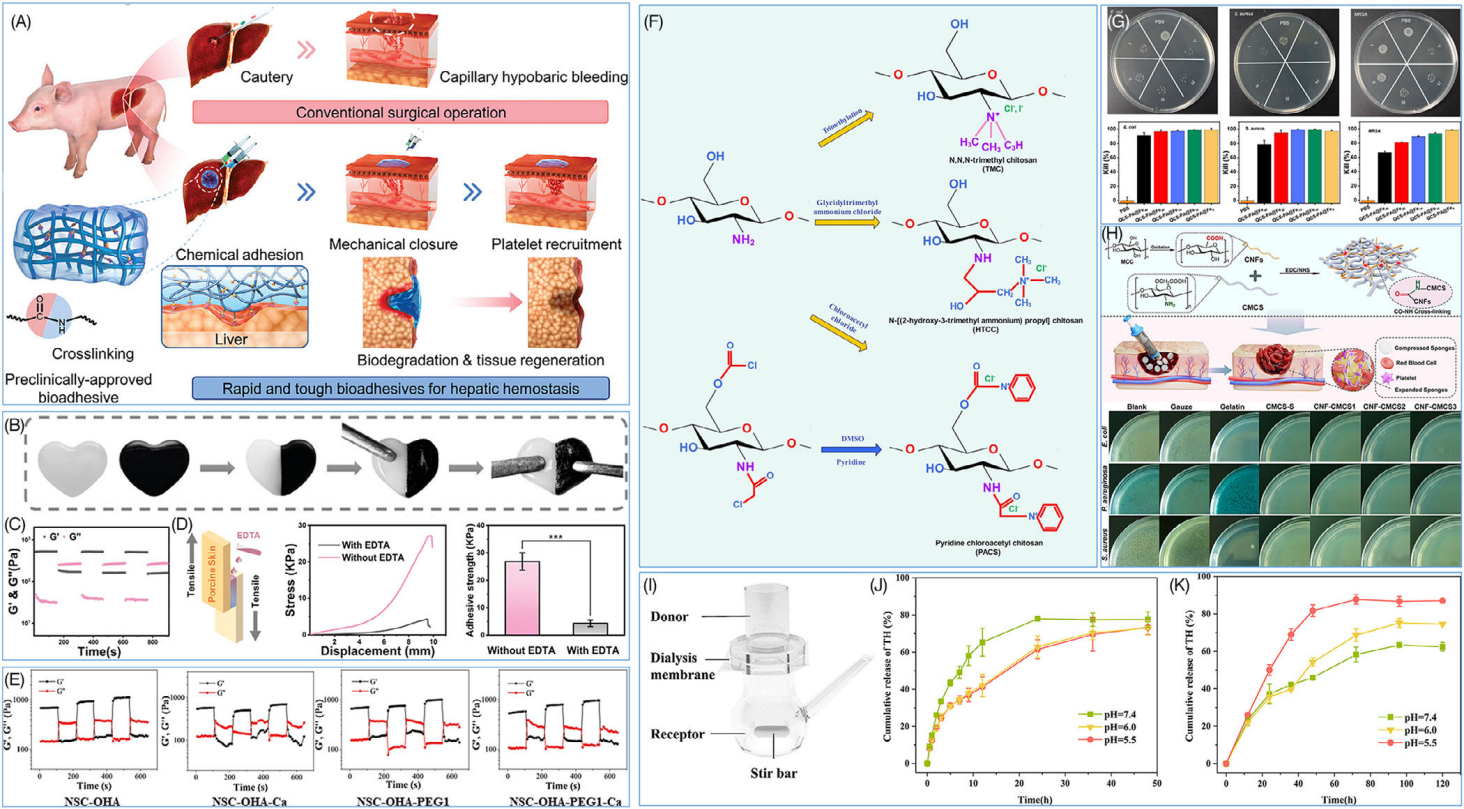
Properties of wound healing materials. (A) Highly tough and biodegradable PEG bioadhesive for hemostasis in porcine liver injury.^116^ Reproduced with permission, © Wiley‐VCH GmbH. (B) Self‐healing process of CaGOD/ZnO. (C) Strain sweep of CaGOD/ZnO under alternate step mode. (D) Shear performance testing of pigskin laps demonstrates that use of EDTA can effectively remove CaGOD/ZnO wound dressings without causing secondary damage to the wound.^120^ Reproduced with permission, © Wiley‐VCH GmbH. (E) Rheological tests of OHA‐containing hydrogel under cyclic strains ranging from 1 to 500%.^121^ Reproduced with permission, © Elsevier Ltd. (F) Synthetic and preparation scheme of common forms of QCS.^57^ Reproduced with permission, © Elsevier B.V. (G) Surface antibacterial activity tests of the QCS‐containing hydrogels.^122^ Copyright © 2021, American Chemical Society (H) Antibacterial and hemostatic properties of CMCS‐containing hydrogels.^123^ Reproduced with permission, © Elsevier Ltd. (I) The structure of the Franz cell used for the drug release test. (J) In vitro release kinetics of TH from solution in PBS at pH values of 7.4, 6.0, and 5.5. (K) In vitro release kinetics of TH from the hydrogels in PBS at pH values of 7.4, 6.0, and 5.5.^124^ Reproduced with permission, © American Chemical Society.

Wound healing materials must possess a highly effective hemostatic capacity, typically requiring a perfect fit between the material and the wound. Hence, the material's flexibility is very important. Huang et al.^116^ developed an injectable hydrogel by mixing tetra‐armed PEG succinimide glutarate (tetra‐PEG‐SG), tetra‐armed PEG amine (tetra‐PEG‐NH_2_) and tri‐lysine, which efficiently sealed bleeding sites, reducing surgical time by 50% and blood loss to 1/15 compared with a commercially α‐cyanoacrylate‐based adhesive, showing great promise as an effective hemostatic agent (Figure 2A). The flexibility of tetra‐PEG hydrogel allows it to adhere tightly to the wound surface, cover any irregular wound spaces, and provide an effective physical barrier to prevent further bleeding.^117^, ^118^ Simultaneously, the porous network structure exhibits a notable high specific surface area to increase the contact area between the material and the wound, thus allowing the increased interaction sites and adhesion of the material.^119^

Aside from its flexibility, the adhesion of the material is crucial for ensuring a tight connection between the material and the tissue, preventing it from detaching or fracturing throughout the healing process.^125^, ^126^ Some natural or synthetic polymers typically contain several hydrophilic groups, including hydroxyl (‐OH),^127^ carboxyl (‐COOH),^128^ amino (‐NH_2_),^129^ and so on, which enables them to build hydrogen bonds with water molecules, resulting in significant improvement in the materials' water absorption and wet adhesion properties.^44^ Simultaneously, the hydrophilic groups may interact with different biomolecules (e.g., proteins, polysaccharides, etc.) in the exudate and tissue fluid of the wound, such as electrostatic interaction and van der Waals force, thereby enhancing the adhesion between the material and the wound.^130^, ^131^ Furthermore, some smart materials can form dynamic and reversible chemical interactions with the wound tissue,^132^, ^133^ such as Schiff base bonds, ester bonds, and so on. These dynamic chemical bonds not only enhance the adhesion strength between the material and the wound but also possess reversibility, indicating they can be automatically removed once the material has achieved its hemostatic effect.^134^, ^135^ This allows for easy removal and replacement of the material, avoiding any additional harm. Cheng et al.^120^ developed an injectable and self‐healing hydrogel (CaGOD/ZnO) using Schiff base cross‐linking of caffeic acid‐modified gelatin (CaG), oxidized dextran (ODex), and zinc oxide (ZnO), which exhibited self‐healing upon breakage (Figure 2B,C) and was designed for safe removal without causing secondary injuries after use (Figure 2D). Similarly, Weng et al.^121^ designed an injectable hydrogel featuring dynamic chemical bonding using water‐soluble N‐succinyl CS, oxidized hyaluronic acid (OHA), Ca^2+^, and PEG. Rheological results demonstrated the OHA‐containing hydrogel's excellent self‐healing properties under cyclic strains ranging from 1 to 500% (Figure 2E).^121^

### 5.3 Multifunctional modification

Traditional monofunctional materials commonly face many obstacles in clinical applications, giving challenges in satisfying multiple therapeutic demands such as bacterial infection,^135^, ^136^, ^137^ tissue regeneration,^129^, ^138^, ^139^ and drug delivery simultaneously.^136^, ^140^, ^141^ These complicated clinical challenges require materials with multiple functions to provide more comprehensive and efficient solutions. The multifunctional modification strategy offers a novel approach to address this problem. Integrating numerous functional groups or active chemicals into a single material platform makes it possible to endow the material with multiple biological properties and achieve synergistic enhancement among diverse functionalities, thus realizing more comprehensive and efficient therapeutic effects. Here are some promising paths for multifunctional modification.^142^, ^143^, ^144^, ^145^

#### 5.3.1 Antibacterial modification

Infection is a major cause of wound healing and tissue regeneration failure. Currently, the standard clinical strategy is still systemic or local antibiotic treatment, but antibiotic resistance greatly reduces the effectiveness of these treatments.^146^ So, polymer compounds with unique chemical structures or functional groups are getting widespread attention due to their antibacterial or bactericidal effects.

CS is a naturally occurring polymer molecule obtained by deacetylation of chitin, typically from the shells of shrimp and crabs.^9^, ^90^, ^147^ With good biocompatibility, CS can degrade in vivo to nontoxic glucosamine.^9^ Due to the trace positive charge carried in its molecular structure, CS can interact with the negatively charged groups on the surface of bacteria, damage the bacterial cell membrane, and lead to the death of bacteria.^148^ CS can be chemically modified to improve its antibacterial properties. Quaternization modification is an effective strategy for increasing the cation density of CS and thus boosting the interaction between CS and bacterial cell membranes (Figure 2F).^147^ Thus, the antibacterial activity of CS is greatly improved. Liang et al.^122^ developed a series of antibacterial hydrogels via dual‐network cross‐link between trivalent iron (Fe^3+^), proanthocyanidin containing phenolic and aldehyde groups, and quaternary ammonium‐modified CS (QCS). The surface antibacterial activity demonstrated QCS‐containing hydrogels' excellent antibacterial performance against Gram‐negative Escherichia coli (>90%), Gram‐positive Staphylococcus aureus (∼80%), and methicillin‐resistant Staphylococcus aureus (>60%) (Figure 2G).^122^ Furthermore, carboxymethylation is also a useful method of modification. The water solubility of CS can be enhanced by adding carboxymethyl groups, hence improving its antibacterial activity in aqueous solution.^149^ Zhou et al.^123^ developed an antibacterial hemostatic sponge by modifying carboxymethyl CS (CMCS) with cellulose nanofibers, which can rapidly absorb water and expands, effectively achieving antibacterial and hemostatic effects (Figure 2H). Overall, these chemical modification techniques not only greatly enhance the antibacterial characteristics of CS, but also give ideas for creating new antibacterial medications, thereby broadening its potential applications in wound healing and tissue regeneration.

#### 5.3.2 Drug carrier modification

Properly designed hydrogels as drug delivery have the potential to significantly improve drug therapy, reduce side effects, and enhance the overall experience for patients.^150^ Hydrogels can be physically or chemically encapsulated with drugs inside and used for controlled drug delivery by regulating the drug release rate.^101^, ^151^ Sustained‐release drugs may prolong the duration of drug effects in the body, decrease the frequency of drug administration, and prevent the drug from reaching its highest concentration in the bloodstream.^152^ This helps to minimize the potential harmful side effects that might occur due to excessive drug concentrations. Additionally, hydrogels can work as a barrier to prevent the degradation or inactivation of drugs in the external environment, thus improving drug stability and biological activity.

Besides, hydrogels can be modified by incorporating specific ligands, such as antibodies, peptides, and glycan chains, to provide active targeted drug delivery.^150^ Through ligand–receptor specific recognition and binding, the modified hydrogel can selectively deliver drugs to target cells or tissues, resulting in precise drug delivery, enhancing efficiency, and minimizing toxicity.^153^, ^154^, ^155^ Meanwhile, hydrogels can also be designed to be smart, responsive materials sensitive to specific in vivo environments,^156^ such as pH, temperature, enzymes, and so on. These responsive hydrogels can trigger drug release based on changes in the microenvironment at the lesion site, resulting in on‐demand drug delivery and improving the precision of drug delivery. Li et al.^124^ developed a tetracycline hydrochloride (TH) sustained‐release platform by creating a hydrogel based on the Schiff base reaction between oxidized sodium alginate (OA) and ethylenediamine‐grafted hyaluronic acid (HA‐EDA). Utilizing the imine bond's hydrolysis in acidic environments and the alkaline nature of chronic wounds, the TH‐loaded OA/HA‐EDA hydrogel achieved sustained release for 120 h, inducing long‐term antibacterial effects (Figure 2I,J,K).^124^

### 5.4 Summary and perspectives

The development of wound healing materials has entered a new era with increasing demands for sophisticated functionalities and superior performance. Current research has established three fundamental criteria for ideal materials: basic performance parameters, functional properties, and practical applications. The essential performance concludes biocompatibility, mechanical properties, hemostatic efficiency, and tissue adhesive properties, which form the foundation for clinical applications.^157^, ^158^, ^159^ Beyond these fundamental requirements, advanced functional properties have emerged as crucial factors, including antibacterial activity,^160^, ^161^, ^162^ controlled drug delivery abilities,^136^, ^141^, ^163^ tissue regeneration properties,^164^, ^165^, ^166^ and smart responsiveness to environmental stimuli (e.g., pH,^167^, ^168^, ^169^ temperature ^170^, ^171^, ^172^). Furthermore, practical considerations such as ease of storage, operational simplicity, cost effectiveness, and scalability have become increasingly significant in translational research.

The evolution of wound healing materials will advance in four strategic directions. The first focuses on innovative material design strategies, encompassing the development of dynamic chemical crosslinking systems,^120^, ^169^ constructing multifunctional platforms with synergistic effects,^115^, ^160^ and exploring smart, responsive materials.^141^, ^172^ The second direction emphasizes performance optimization, particularly in enhancing hemostatic efficiency, strengthening tissue adhesion,^129^, ^157^ controlling material degradation kinetics,^129^, ^173^ and achieving precise drug delivery.^136^, ^141^ The third strategic focus centers on clinical translation, necessitating the establishment of standardized evaluation protocols, optimization of scale‐up manufacturing processes, cost reduction strategies, and comprehensive clinical trials.^174^, ^175^ The fourth direction involves expanding applications into emerging fields, such as minimally invasive surgery, extreme environment applications (e.g., aerospace medicine), personalized hemostatic solutions, and intelligent monitoring systems.

These multifaceted challenges and opportunities drive hemostatic material research and development paradigm shifts. Next‐generation hemostatic materials are expected to revolutionize trauma treatment and tissue repair through interdisciplinary collaboration and integration of academia‐industry partnerships. The continuous emergence of innovative technologies and theoretical frameworks will undoubtedly facilitate breakthrough advances in healthcare applications, ultimately leading to improved patient outcomes and expanded therapeutic possibilities.

## 6 DIFFERENT FORMS OF WOUND HEALING MATERIALS

Different forms of wound healing products possess unique characteristics, and the comprehensive selection of suitable materials can greatly improve efficiency. Materials such as gauze, hydrogel, powder, and sponge have been developed. This section will provide an in‐depth review of several materials with the potential for hemostasis and wound healing in clinical applications.

### 6.1 Gauze

Gauze, a traditional and widely used material, exhibits excellent water absorption and permeability, efficiently absorbing blood and accelerating coagulation. The porous structure of gauze promotes the aggregation of red blood cells and platelets, accelerating the concentration of coagulation factors and the coagulation cascade. Oxidized regenerated cellulose, the main component of common hemostatic gauze, possesses biocompatibility, degradability, and antimicrobial characteristics.^176^ Optimal performance of hemostatic gauze can be achieved by controlling the oxidation degree of cellulose and the weaving density during preparation. Nevertheless, an excessive amount of oxidation may reduce the material's strength. Despite its advantages, gauze has limitations regarding severe bleeding and may lead to secondary bleeding or foreign body reactions due to fiber detachment.

**FIGURE 3:**
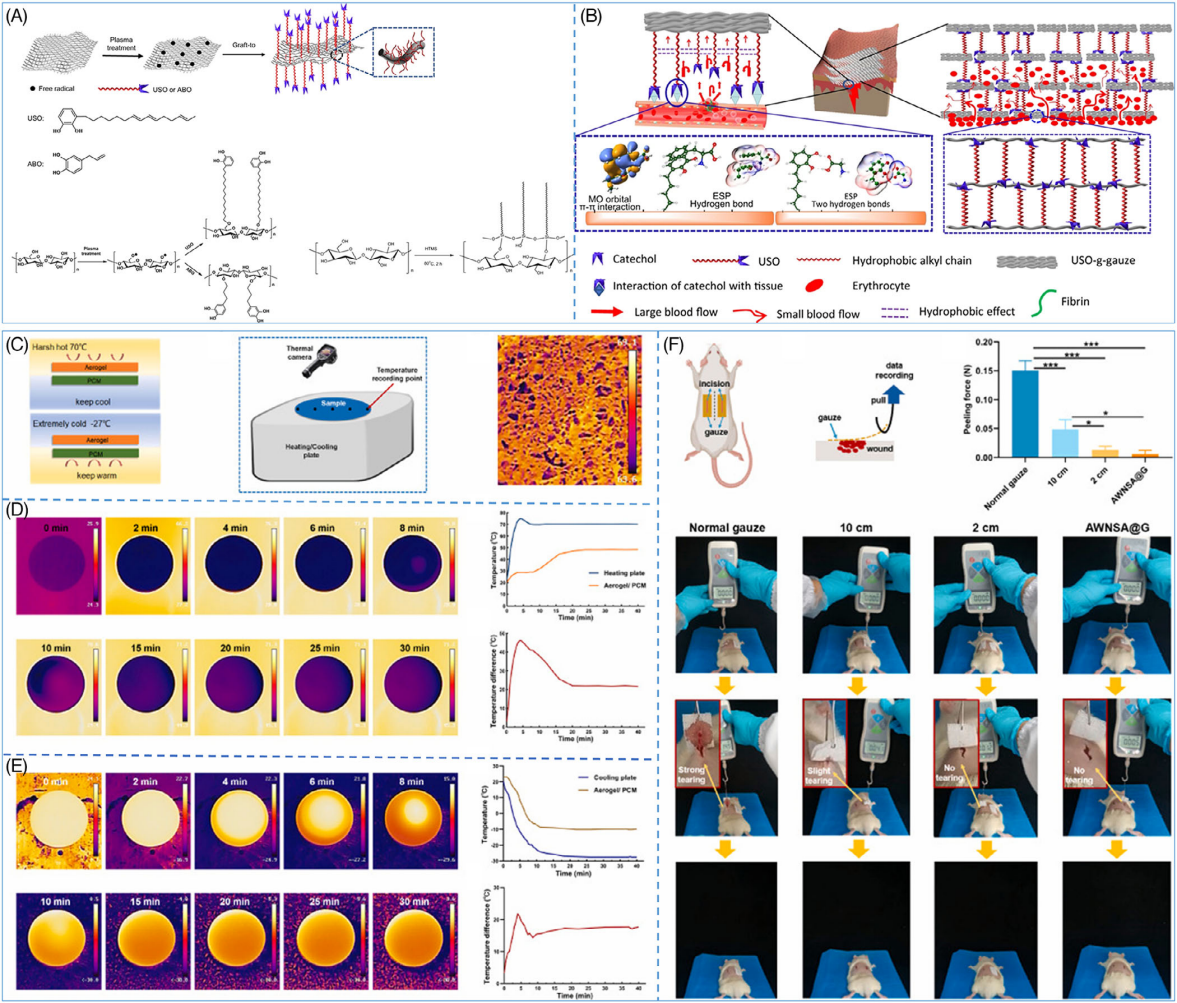
Synthesis and characterization of gauzes. (A) Schematic diagram of grafting chemicals containing catechol groups onto cotton fiber. (B) Mechanism of hemostasis of USO‐g‐gauze.^159^ Reproduced with permission, © The Author(s). (C) Schematic diagrams of LBL structures assembled with nano‐aerogel layers and phase change material layers in extreme environments and LBL structures tested on a heated/cooled plate. (D) Infrared thermography images, time–temperature, and time–temperature difference curves of samples on a heating plate at specified time points. (E) Infrared thermography images, time–temperature, and time–temperature difference curves of the sample on the cooling plate at specified time points. (F) Rat back gauze peeling test.^178^ Reproduced with permission, © Elsevier B.V.

Numerous efforts have been made to enhance the hemostatic efficacy of gauze; wherein surface modification is an effective means. He et al. generated a highly effective hemostatic gauze by modifying the surface with the catechol chemical‐based 1,2‐benzenediol‐3‐(7,9,13‐pentadecatrienyl) (USO), which integrated the wet tissue adhesion of the catechol moiety, the hydrophobicity of the long alkyl chains, and the absorbency and breathability of the cotton fibers (Figure 3A).^159^ The gauze demonstrated remarkable hemostatic performance in a porcine femoral artery injury model. The catechol moiety rapidly adhered to the skin tissue by noncovalent bonds, creating a barrier surrounding the wound. The hydrophobic interactions between the long C15 chains prevented blood from seeping into the upper gauze layer, resulting in successful hemostasis (Figure 3B). Furthermore, the noncovalent adhesion between the catechol and the tissue can be broken by a slight peeling force, minimizing secondary bleeding upon gauze removal.^159^ Similarly, taking advantage of the fact that the catechol moiety can bind noncovalently to tissues and that the phenolic hydroxyl groups and hydrophobic chain segments of tannic acid (TA) immobilize thrombin on the surface of cellulose gauze, Ye et al.^177^ developed a composite gauze Gau@TA/Thr based on thrombin, which not only improved the hemostatic ability of the gauze but also effectively avoided the thrombosis problem caused by the excessive release of thrombin in previous thrombin‐based materials. In addition to catechol moieties, some nanomaterials can also be used as coatings for gauze to satisfy hemostasis in harsher environments. Jia et al.^178^ developed a layer‐by‐layer structured gauze (AWNSA@G) by spraying silica nano‐aerogel onto the surface of the gauze. The extremely low thermal conductivity of the aerogel enables the gauze to maintain the relative stability of the wound microenvironment in both extreme cold (−27°C) and heat (70°C) conditions (Figure 3C–E). In a rat back gauze peeling test, AWNSA@G exhibited significantly lower peeling force than ordinary gauze, and it did not cause any tearing to the wound during the removal process (Figure 3F).^178^

Although hemostatic gauze has been used extensively in clinical settings for its ability to stop bleeding, it still has several limitations. Researchers are focusing on developing novel functionalized hemostatic gauzes to overcome these drawbacks. However, current studies on these novel gauzes concentrate on surface modifications or coatings. Future studies should explore the potential of manipulating the individual components of the gauze. For instance, by leveraging 3D printing technology, researchers may integrate advanced materials into the gauze skeleton to obtain new hemostatic gauze products that can be more easily mass‐produced and applied.^179^

### 6.2 Hydrogel

Hemostatic hydrogels are an unusual type of topical hemostatic materials, which can quickly stop bleeding and protect wounds by in situ swelling and expansion.^180^ The core characteristic of hemostatic hydrogels is their ability to adhere to tissue through the reaction between the reactive groups present in the hydrogel and the amino groups of proteins in the tissue, thus enabling it to seal the wound and accelerate the hemostatic process efficiently.^108^

**FIGURE 4:**
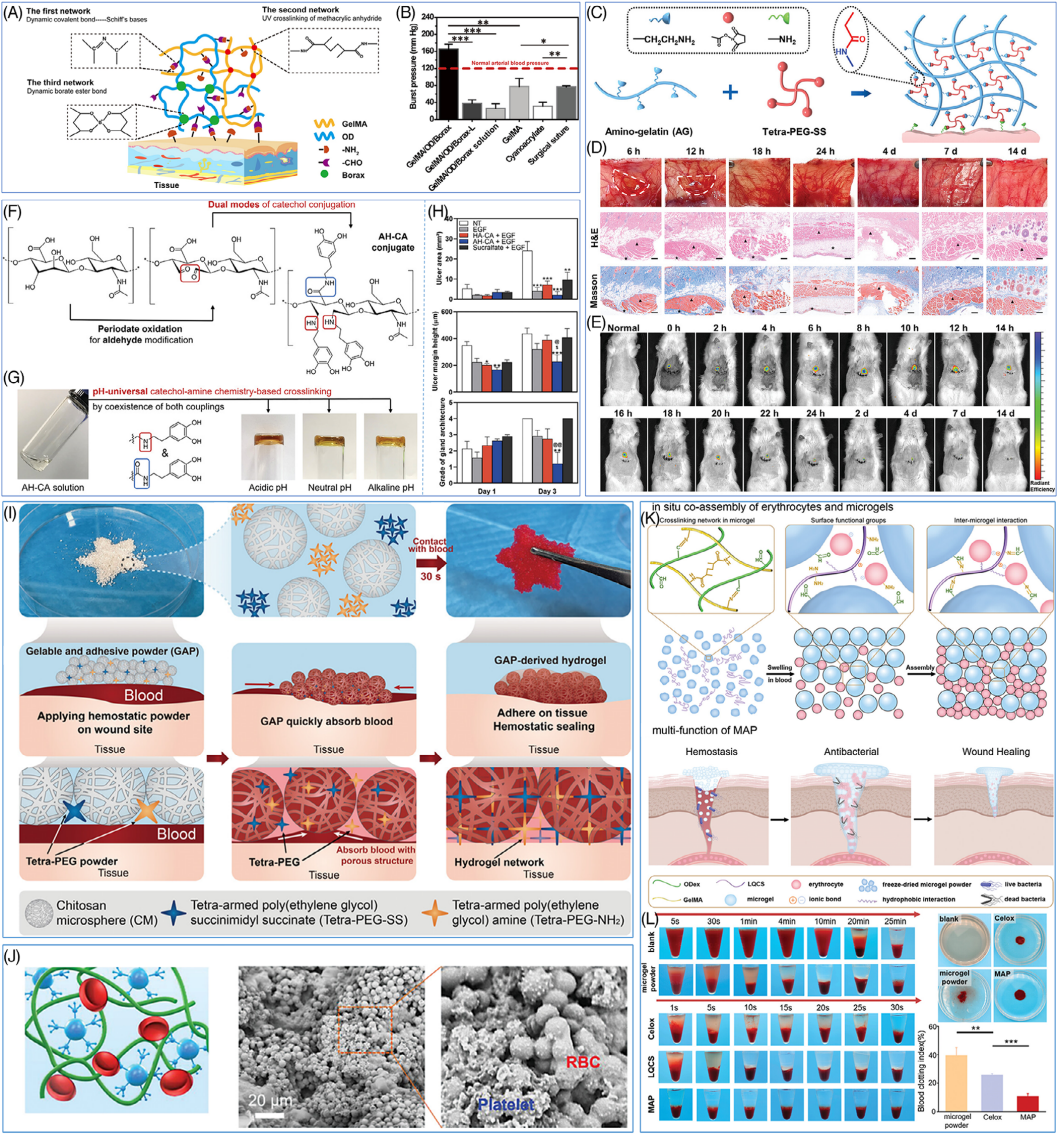
Preparation and characterization of hydrogels. (A) Triple crosslinking network structure of GelMA/OD/Borax. (B) Burst pressure of GelMA/OD/Borax.^181^ Reproduced with permission, © Elsevier B.V. (C) Schematic diagram of the interaction between AG–PEG hydrogel and tissue. (D) Inflammatory response to subcutaneous implantation of AG–PEG hydrogel. (E) In vivo degradation of AG–PEG hydrogel.^129^ Reproduced with permission, © Wiley‐VCH GmbH. (F) Preparation process of oxidized hyaluronic acid and AH–CA. (G) Formation of AH–CA hydrogel at different pH conditions (acidic: 2.5, neutral: 6.8, and basic: 7.5). (H) Superior proulcer recovery of AH–CA in a mouse gastric model.^184^ Reproduced with permission, © Wiley‐VCH GmbH. (I) Design and mechanism of the GAP.^185^ Reproduced with permission, © Wiley‐VCH GmbH. (J) Red blood cell adhesion of ColTA.^186^ Reproduced with permission, © Wiley‐VCH GmbH. (K) Mechanism of MAP coassembly with erythrocytes. (L) In vitro hemostatic characterization of blank, microgel powder, Celox powder, LQCS powder and MAP.^187^ Reproduced with permission, © Wiley‐VCH GmbH.

Photo‐induced cross‐linking is a common method to prepare hemostatic hydrogels. During this process, the precursor fluid of the hydrogel undergoes rapid crosslinking and solidification when exposed to UV light or infrared light within seconds to minutes. The mechanical strength of the hydrogel can be controlled precisely to suit various bleeding situations by manipulating the concentration of the hydrogel precursor liquid and the quantity of cross‐linking agent. Gelatin methacryloyl (GelMA) is commonly used as a component in photocrosslinking curing. Chen et al.^181^ successfully synthesized GelMA/OD/Borax hydrogels with a triple crosslinking network structure by mixing GelMA with oxidized dextran (OD) and borax, and then performing photo‐induced crosslinking (Figure 4A). The GelMA chain undergoes a reaction with the methacrylic anhydride group to establish the first cross‐linking network. The remaining amino group of GelMA reacts with the aldehyde group of OD to form a Schiff base bond, which constitutes the second cross‐linking network. Additionally, the dynamic borate ester bond between borax and OD forms the third cross‐linking network. Furthermore, the aldehyde group present on the remaining OD chain reacts with the proteins in the tissue to augment the adhesion of the hydrogel, allowing the hydrogel to attach to the moist tissue surface firmly. The GelMA/OD/Borax hydrogel demonstrates exceptional mechanical strength and adhesion due to its triple network structure. It can endure pressures of up to 165.53 mmHg, which is much greater than the typical systolic pressure, while effectively sealing the bleeding area (Figure 4B). The significant mechanical strength indicates that hydrogel can effectively withstand blood pressure, highlighting its potential use as a hemostatic and tissue adhesive in visceral hemostasis. Photo‐crosslinked hemostatic hydrogels offer numerous benefits but also present significant challenges. Their preparation and application require specialized light sources, limiting their use in certain settings. Prolonged light exposure risks phototoxic tissue damage due to harmful chemical byproducts. Moreover, the limited penetration depth of light restricts their utility in managing deep wounds or tubular bleeding. Despite efforts to optimize photo initiator safety, toxicity risks persist, particularly in long‐term applications, necessitating extensive clinical studies to establish their long‐term safety for widespread use.^53^, ^182^, ^183^

In addition to photo‐induced cross‐linking hydrogels, there has been a rise in the development of injectable hydrogels that can be directly applied to wounds or specific areas of the body through a simple injection and can self‐cure without the requirement of outside irradiation or the addition of extra chemical cross‐linking agents, which simplifies the clinical procedure and minimizes patients' discomfort.^129^, ^134^, ^181^ Wang et al.^129^ produced a novel injectable hydrogel by aminomethylating the gelatin molecules and enhancing the amino content of the gelatin molecules. The gelatin molecule underwent amination to enhance the amine content of gelatin (AG), which increased its reactivity with tetra‐armed poly(ethylene glycol) succinimidyl ester (tetra‐PEG‐SS), enabling a faster reaction and the formation of a stable chemical bond between AG and PEG‐SS (Figure 4C). This rapid reaction rate allowed AG–PEG glue to stop bleeding in emergencies immediately, helping to control bleeding and promote wound healing. Additionally, hydrogel's rapid biodegradation within 24 h and minimal inflammatory response from its biocompatible degradation products offered significant advantages, allowing the hydrogel to effectively achieve hemostasis and protect wounds without necessitating surgical removal, reducing the risk of secondary interventions (Figure 4D,E). The lack of immunological or inflammatory complications is crucial for the hydrogel's safe long‐term use.^188^ However, the cost of PEG derivative is a major obstacle to its widespread clinical application. The complex and costly process of synthesizing, purifying, and functionalizing PEG is particularly challenging for large‐scale production and low‐cost medical needs.

Mimicking the adhesion mechanism of mussels or other marine organisms to promote the adhesion between hydrogels and biological tissues is a common means of preparing biomimetic hydrogels.^189^, ^190^ The peduncle glands of mussels can secrete a unique protein known as “mussel adhesive protein,” which possesses a strong adhesive property and enables mussels to adhere firmly to objects. The strong adhesion of mussel mucin is mainly due to the catechol groups, which can be oxidized to quinone at high pH levels and further cross‐linked to produce a polymer mesh structure that enables strong adhesion.^191^, ^192^ However, the application of mussel mucin is restricted by its sensitivity to pH. An et al.^184^ proposed a pH‐independent multifunctional hydrogel AH–CA by partially oxidizing hyaluronic acid to introduce aldehyde groups and increase reactive sites. This hydrogel had the potential for pH‐independent drug delivery to acidic tissues, pH‐independent tissue sealing, and hemostasis applications (Figure 4F,G). In a mouse gastric drug delivery model, AH–CA loaded with EGF exhibited excellent adhesion to the gastric surface for protecting gastric wounds. Besides, it aided the delivery of EGF to acidic gastric tissues, thereby promoting gastric ulcer healing (Figure 4H).^184^ This study showed the potential of catechol‐based hydrogels with pH‐independent adhesion properties for biomedical applications and offers useful insights for the advancement of novel biomaterials.

Hemostatic hydrogels can be given additional properties, such as self‐healing, antimicrobial, and responsive properties, to enhance the wound healing process. Responsive hydrogels can respond to specific biological signals in vivo (pH,^193^ glucose,^140^ enzymes,^194^ etc.). For instance, the dynamic bonding between glucose and a hydrogel can be achieved by grafting a phenylboronic acid group to the hydrogel, which leads to responsive drug release, providing unique advantages in diabetic wound healing.^195^ Antimicrobial properties can be achieved by adding antimicrobial components, such as some drugs ^196^ (antibiotics, tetracycline, etc.), polymers (QCS,^147^ antimicrobial peptides,^197^ etc.), and nanoparticles (Ag,^198^ ZnO,^120^ etc.). These antimicrobial components can continually act to prevent bacterial growth at wound sites, thereby reducing the risk of infection and accelerating wound healing. Self‐healing can be realized by introducing components with dynamic chemical bonds, such as disulfide bonds and imine bonds.^199^, ^200^ When the hydrogel is broken, these dynamic chemical bonds can be broken and reformed so that the hydrogel can repair the broken part independently and restore its original structure and function. These functionalized hydrogels not only display obvious advantages in promoting wound healing, reducing infections, and improving patient comfort but also demonstrate the innovation complexity of advanced materials science applications in the medical field. Therefore, functionalized modifications can confer excellent performance of hydrogels in the wound healing process, but it is still challenging to design the structure of hydrogels further to match better the four phases (hemostasis, inflammation, proliferation, and remodeling) of wound healing.

### 6.3 Powder

Hemostatic powder is a type of powdered hemostatic agent, which can be simply applied to the wound. Hemostatic powder usually consists of porous particles with a high specific surface area, which enables hemostatic powder's superior ability to absorb fluid at the wound.^201^ When the hemostatic powder is applied to the bleeding wound, the surface and pores of the powder will rapidly absorb the blood seeping out of the wound, thereby locking the blood in the microstructure of the particles. This process not only effectively prevents further blood leakage but also restricts the blood to the localized area of the wound, thereby preventing massive blood loss.^1^, ^109^, ^202^ Zhang et al.^185^ reported a new gelable and adhesive powder (GAP) in their study. This powder can form a physical barrier and bioactive microenvironment at the wound site to achieve rapid hemostasis. The GAP powder consisted of three main components: CS microspheres (CM), tetra‐PEG‐NH_2_, and tetra‐PEG‐SS. The macroporous structure of CM rapidly absorbs blood, promoting gelation. Then, the cross‐linking of tetra‐PEG‐NH_2_ and tetra‐PEG‐SS in a humid environment forms a stable gel network that immobilizes the blood and prevents further loss (Figure 4I). Besides, the positive charge of CM stimulates the coagulation process and promotes blood clot formation.^185^ This fabrication of hemostatic powder using porous microspheres compounded with hydrogel precursor powder provides valuable ideas and practical experience for the development of new hemostatic powders.

The hemostatic mechanism of hemostatic powder not only depends on the physical adsorption of porous microspheres but also involves the regulation of the coagulation cascade by chemical procoagulant components. Many hemostatic powder materials contain active ingredients, including platelet, collagen, and Ca^2+^, that directly participate in and accelerate the coagulation process.^56^, ^203^, ^204^ Shang et al.^186^ discovered a new type of powder called collagen/TA natural hemostatic binder powder (ColTA), which uses the noncovalent interactions between collagen type II (Col‐II) and TA in aqueous solution to achieve water‐sensitive and ultra‐fast in situ self‐gelation properties. This ColTA powder can boost red blood cell adhesion, hence promoting hemostasis (Figure 4J).^186^ However, it is crucial to consider the dosage of coagulant active ingredients during the design of hemostatic powders. While increasing the ratio of coagulant active ingredients in the material might greatly promote the hemostatic process, an excessive amount of active ingredients may result in undesirable reactions like thrombosis, which may be a risk to the patient's health.

Some compounds have exhibited coagulation‐promoting properties in the design of hemostatic powders, in addition to the traditional bioactive ingredients, giving new ideas for addressing the thrombosis issue. Li et al.^187^ described a new composite hemostatic powder (MAP) composed of porous OD/gelatin methacrylate (ODex/GelMA) microgel powders and long‐chain alkyl QCS (LQCS) cross‐linker powder, and LQCS is the key to promote coagulation (Figure 4K,L). The main chain of the LQCS is positively charged while the surface of the erythrocyte membrane is rich in negatively charged sialic acid and other groups, which generates a strong electrostatic interaction, allowing for the selective adsorption and concentration of erythrocytes on LQCS chain. Additionally, the lipid bilayer of the erythrocyte membrane contains numerous hydrophobic groups, which can interact with LQCS and other hydrophobic groups to promote erythrocyte adherence further. The erythrocytes on the surface of MAP can accelerate the platelet aggregation and activation process, facilitate the conversion of plasminogen to thrombin, and serve as a framework for the creation of a fibrin clot, thus accelerating the hemostatic process. In addition, the adsorbed erythrocytes can also fill the wound defect and form a tight wound closure together with the clot to prevent secondary bleeding.^187^, ^205^ Compared with traditional bioactive components, LQCS and other chemical crosslinkers have the advantages of low cost, stable performance, and high safety. This chemical crosslinker‐mediated red blood cell adsorption strategy provides a new way to solve the problem of thrombus caused by hemostatic powder. However, due to the small size of hemostatic powders, they are easy to circulate along the blood vessels and may cause blood clots somewhere in the body. Therefore, the degradation time of styptic powder is reasonably designed to solve the problem of thrombus further.

### 6.4 Sponge

The hemostatic sponge is a porous and three‐dimensional material designed for effective hemostasis. Its porous structure allows it to rapidly absorb fluid from the wound and fill the wound cavity through swelling, thus creating constant pressure on the wound and effectively stopping bleeding.^206^ Besides, the pore wall surface can absorb coagulation factors and platelets, increasing the specific surface area for the coagulation reaction and facilitating prompt hemostasis. Due to its excellent liquid absorption, compressive hemostatic effect, and ability to fill the wound cavity, the hemostatic sponge is particularly suitable for complex wounds, such as deep oozing and sinus wounds that are difficult to stop bleeding by direct compression.^202^, ^207^

**FIGURE 5:**
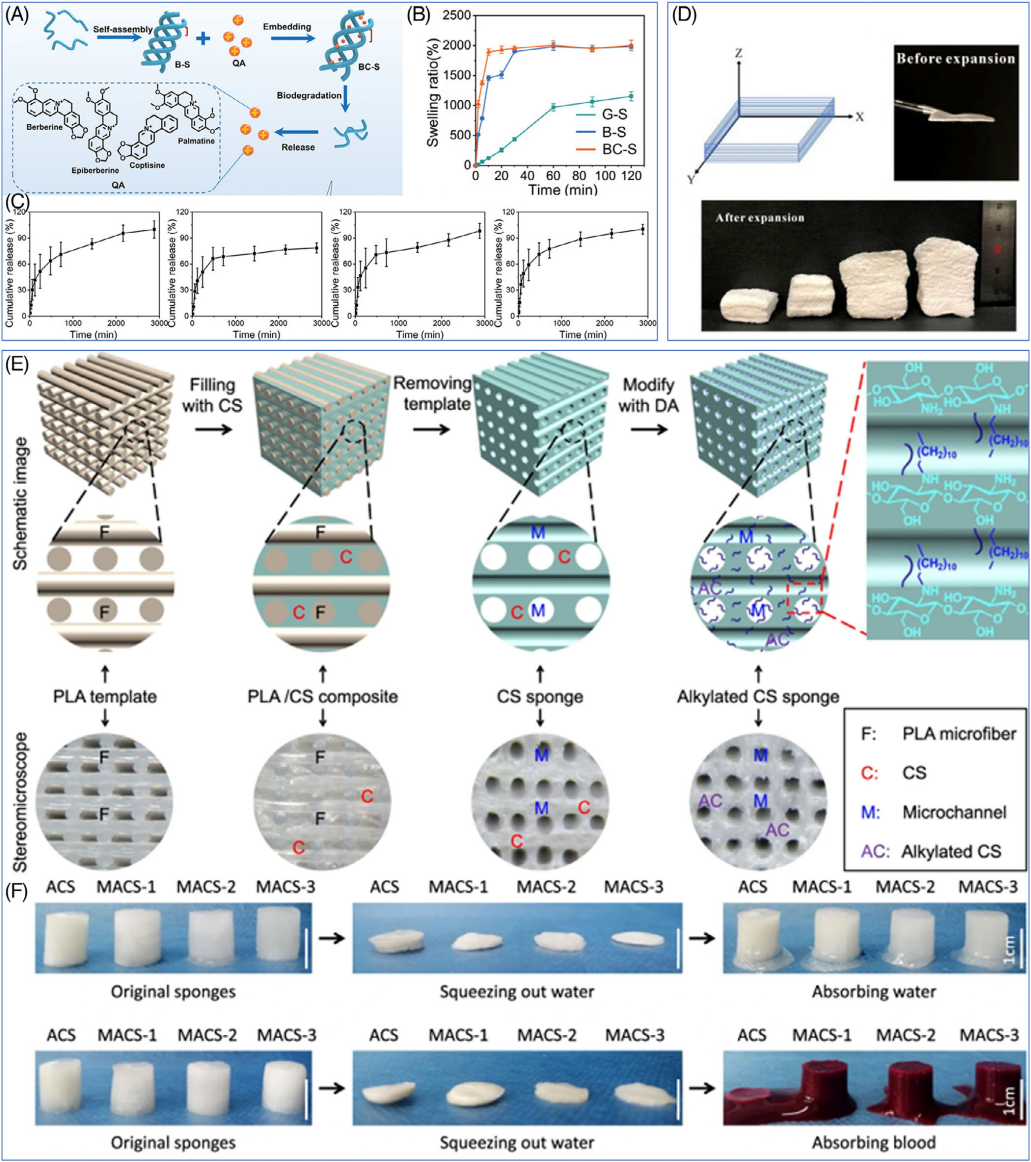
Structure and preparation of sponges. (A) Synthesis of BC‐S and QA release mechanism. (B) Water absorption and expansion of BC‐S. (C) Cumulative release profiles of palmatine hydrochloride, epiberberine, cotinine hydrochloride, and berberine hydrochloride.^137^ Reproduced with permission, © Wiley‐VCH GmbH. (D) Schematic shows the direction of 3D CS/PVA nanofiber sponge and the morphological changes of the CS/PVA nanofiber membrane before and after expansion.^209^ Reproduced with permission, © Elsevier Ltd. (E) Stereomicroscopic images of PLA microfiber templates, CS/PLA composites, microchannel CS sponges and MACS sponges. (F) Macro photographs of water‐triggered and blood‐triggered shape recovery in ACS and MACS‐1/2/3.^210^ Reproduced with permission, © The Author(s).

Hemostatic sponges can be synthesized mainly in two ways: (1) lyophilization, in which the precursor solution or hydrogel is lyophilized and water is removed in situ to get sponges with a uniform pore‐like structure;^137^, ^208^ (2) poration, in which additional components help to create a porous structure. Among these, lyophilization is now the most common method for hemostatic sponges (e.g., gelatin sponges) extensively utilized in clinical practice due to its simplicity and efficacy, and it is also the favored synthesis method for the new hemostatic sponges. Inspired by the theories of traditional Chinese medicine, Li et al.^137^ developed a novel multilayer porous hemostatic sponge (BC‐S). The active ingredients of two traditional Chinese medicines, Bletilla striata (BS) and coptidis rhizoma (CR), were utilized to create the sponge.^137^ The cavity triple‐helical polysaccharide BSP from BS and the quaternary alkaloid QA from CR were extracted, and the sponges were subsequently lyophilized after the two components were mixed (Figure 5A). The unique layered porous structure of the BC‐S hemostatic sponge demonstrates excellent hemostatic capabilities and wound healing effects. On the one hand, the high porosity of the sponge gave it a strong liquid‐absorbing ability (2002.50 ± 88.11%), which was conducive to the rapid absorption of wound exudate, concentration of coagulation factors, and acceleration of the hemostatic process (Figure 5B). On the other hand, the sponge can be utilized as slow‐release drug delivery to achieve fast hemostasis while continuously delivering antibacterial components into the wound to prevent wound infection and stimulate tissue healing (Figure 5C). Hemostatic sponge has excellent l fluid absorption performance and shows significant prospect in hemostatic application through the balanced combination of traditional Chinese medicine principles. However, these sponges have certain limitations, including unregulatable pore sizes and shape, poor mechanical properties (predominantly tensile strength), prolonged reaction times, and challenges in degradation time control.

In addition to the lyophilization method, the pore formation with added components is another important way to create porous hemostatic sponges. Based on the various mechanisms for creating pores, adding components to create pores primarily consists of two methods: foaming and sacrificial template. The foaming method involves the introduction of gas into the material, causing it to expand and form a porous structure.^211^ For example, sodium borohydride can react with water to release hydrogen gas, and the researchers immersed a CS‐polyvinyl alcohol nanofibers film prepared by electrospinning into sodium borohydride solution and triggered the foam reaction (Figure 5D).^209^ As a result, the two‐dimensional nanofiber film effectively expanded into a three‐dimensional sponge with a high porosity level. In contrast to the traditional foaming process, this method was simple without requiring extreme conditions of high temperature and pressure. Moreover, it successfully preserved the nanofiber structure of the material, giving the sponge excellent mechanical properties and biological characteristics. In the sacrificial template method, a removable template was introduced into the material and removed by selective dissolution or pyrolysis to obtain porous sponges with uniform and controllable porosity.^139^, ^212^ Taking the preparation of microchannel alkylated CS sponge (MACS) as an example, the researchers first prepared CS/PLA composite scaffolds as sacrificial templates by using 3D printing technology, then submerged them in NaBH_3_CN solution and dissolved the PLA to obtain the CS skeleton after the solution had fully penetrated the scaffolds' pores, and finally obtained MACS sponges with interconnecting microchannel structures after the alkylation modification (Figure 5E).^210^ The sacrificial template method offers an advantage over the foaming method by allowing precise regulation of the sponge's porosity and pore size distribution, which results in excellent shape memory properties and broad potential applications in the field of hemostasis and wound healing (Figure 5F). Regardless of whether the foaming approach or the sacrificial template method applies, the additional components often consist of shirting chemicals, such as sodium borohydride, whose metabolic kinetics and biosafety in vivo are still not well understood, and there are still safety concerns associated with its clinical application. Hence, developing novel nontoxic and biodegradable porous additives is essential for optimizing the future manufacturing process of hemostatic sponges.

Designing multifunctional hemostatic sponges is an increasingly popular trend in this field. Along with their basic hemostatic function, researchers are also committed to giving the sponges additional properties, such as antimicrobial, promoting angiogenesis and accelerating wound healing.^213^ However, some issues still need to be resolved before hemostatic sponges can be used in real life. Expansion of absorbed fluid is the basic mechanism for hemostatic sponges to play the role of compression hemostasis. Nevertheless, it is important to recognize that excessive compression may have adverse effects on the nerves, resulting in pain and distress for the patient and potentially leading to secondary injury. Consequently, precise regulation of the sponge's expansion rate is essential in designing the new hemostatic sponge. Additionally, the removability of hemostatic sponges is a challenge. Removing nondegradable sponges following surgery will usually break the formed hematoma, which may result in secondary hemorrhage, prolong the healing process, and raise the risk of infection. Although biodegradable sponges can avoid the need for a second surgery, their degradation rate may interfere with normal tissue repair if it does not match the healing process. For instance, too rapid degradation may result in clot dislodgment, while too slow degradation may induce a foreign body reaction and ultimately delay wound healing. Consequently, a key to achieving the clinical applications of biodegradable hemostatic sponges is the precise regulation of their degradation behavior to align with the timeline of wound healing.

### 6.5 Summary and perspectives

Various wound healing materials have evolved significantly to meet diverse clinical needs, each offering unique advantages while facing distinct challenges (Table 3). Gauze remains a fundamental and widely used option due to its simplicity and cost effectiveness, though its functionality is being enhanced through surface modifications and advanced manufacturing techniques.^214^, ^215^ Hydrogels represent a more sophisticated approach, offering superior tissue adhesion and controllable properties, but face challenges in production costs and long‐term safety validation.^216^, ^217^ Powder‐based materials are excellent in their ease of application and rapid action, although careful consideration must be given to their potential systemic effects.^218^ Sponges provide excellent wound cavity filling and fluid absorption capabilities, making them particularly valuable for complex wounds despite challenges in controlling their structural properties.^219^ Future research should focus on developing materials that can adapt to different wound environments while maintaining optimal therapeutic effects throughout the healing process. This will require continued innovation in material design, processing techniques, and clinical evaluation methods.

**TABLE 3:** Summary of materials in different forms.

Material type	Advantages	Disadvantages	Future directions
Gauze	Excellent water absorption and permeability Promotes RBC and platelet aggregation Simple to use and cost effective Widely used in clinical settings	Limited efficacy for severe bleeding Risk of secondary bleeding Possible foreign body reactions from fiber detachment Requires regular replacement	Develop novel functionalized surface modifications Integrate advanced materials using 3D printing Improve gauze skeleton components Develop products more suitable for mass production
Hydrogel	Rapid hemostasis and wound protection Good tissue adhesion Controllable mechanical strength Good biodegradability Injectable properties	Photo‐crosslinking requires special light sources Limited light penetration depth High cost of some raw materials pH sensitivity issues Long‐term safety needs further validation	Develop new self‐healing materials Enhance antimicrobial properties Develop responsive hydrogels Optimize structure to match wound healing phases Reduce production costs
Powder	Easy to apply High specific surface area Excellent fluid absorption Rapid physical barrier formation	Risk of embolism through blood circulation Difficult to control active ingredient dosage Potential thrombosis risk Challenging degradation time control	Optimize degradation time design Develop new chemical crosslinkers Address thrombosis issues Improve biosafety
Sponge	Porous 3D structure Excellent fluid absorption Can fill wound cavities Suitable for complex wounds Drug delivery capability	Difficult to regulate pore size and shape Poor mechanical properties Long reaction time Challenging degradation control Removal may cause secondary bleeding	Develop nontoxic biodegradable pore‐forming additives Precise regulation of expansion rate Optimize degradation behavior Develop multifunctional hemostatic sponges Improve mechanical properties

## 7 CONCLUSION AND PROSPECT

Hemostasis is an indispensable part of wound healing. With a deepening investigation into the mechanisms of active ingredients in hemostatic materials, researchers have proposed numerous new strategies for optimizing these materials' composition and fabrication processes. It is anticipated that superior comprehensive performance in terms of hemostatic rate, durability, and biocompatibility can be achieved through the rational design of the types and quantities of components in hemostatic materials, along with the implementation of chemical alterations and formulation optimizations. Furthermore, investigating innovative composite hemostatic materials has broadened potential opportunities for advancement. However, transitioning hemostatic materials from laboratory research to clinical application presents multiple challenges.

The following aspects will be the primary focus of hemostatic material research in the future.

1. *Chemical synthesis and processing technology*: The key to the widespread application of hemostatic materials in the future is the development of a faster and simpler synthesis route, which will reduce production costs and increase material yield. Furthermore, the synthesis method should adhere to the principle of green chemistry and employ environmentally friendly and cost‐effective raw materials to minimize the environmental impact.
2. *Simulation of the physiological hemostatic process*: The design of new hemostatic materials that can participate in or facilitate the physiological hemostatic process is a promising research direction. For instance, hemostatic materials that facilitate the contraction of blood clots and improve their stability can be produced to enable timelier and more efficient hemostasis. Furthermore, developing smart bioactive materials with biomimetic natural hemostatic components (e.g., platelets and fibrin) will provide fresh ideas for developing new hemostatic materials.^206^, ^220^
3. *Expanding clinical transformation and application*: Despite the development of numerous hemostatic materials, only a few have been able to enter clinical trials. Although hemostatic materials derived from humans or animals have been employed in clinical trials, there are potential safety hazards, including immune rejection and pathogen infection. Conversely, hemostatic materials composed of straightforward FDA‐approved chemicals are more likely to be approved and commercialized and possess an inherent advantage in clinical translation.^116^
4. *Development of multifunctional hemostatic materials*: Integrating hemostatic function with diagnostic monitoring, therapeutic repair, and so on, is a promising direction for hemostatic materials in future. For instance, we can generate intelligent materials that incorporate real‐time monitoring of physical and chemical parameters (e.g., pH, electrical properties) and hemostatic function to enable dynamic assessment of wound status.^221^, ^222^ We can also develop hemostatic materials integrating the infection monitoring and antibacterial functions to effectively prevent wound infection. Additionally, we can create hemostatic materials that can facilitate the repair of internal organs and accelerate the healing of deep tissue wounds.^188^, ^223^ Introducing these multifunctional materials will significantly broaden the application domains of hemostatic materials and enhance the clinical therapeutic effect.
5. *Optimization removable/degradable behavior*: After completing the hemostatic goal, hemostatic materials must be either removed from the body or degraded. Therefore, optimizing the material's design necessitates enhancing its degradability and removability. Ideally, removable hemostatic materials should be able to be removed smoothly without causing secondary bleeding and pain. In addition, the hemostatic materials should require good biocompatibility of degradation products and compatible degradation rate with tissue healing process, and the degradation process should not trigger a thrombotic inflammatory reaction.^224^, ^225^
6. *Enhance practical performance*: The storage stability, portability, and other practical characteristics of hemostatic materials must be carefully considered in various application scenarios (e.g., military rescue). For instance, the practical requirements of first aid in the field will be more effectively addressed by developing hemostatic materials that are both simple to transport and can be stored for an extended period in extreme environments (high/low temperatures).

In summary, future research on hemostatic materials should not be restricted to enhancing the hemostatic effect; instead, it should concentrate on the expedition of clinical translation and application. By continuously expanding the practicality, improving the removable/degradable performance, simulating the physiological hemostatic processes, developing multifunctional materials, and optimizing the chemical synthesis and processing procedures, we can accelerate the development of a new type of hemostatic materials with integrated rapid hemostasis, efficient repair, and monitoring functions. This will significantly promote the broad application in clinical treatment and benefit patients.

## AUTHOR CONTRIBUTIONS

Xing Wang designed and supervised the study. Zhengyuan Liu and Junnan Xu collected the data, wrote the manuscript drafting, and designed the figures and tables. All authors read and approved the final manuscript.

## CONFLICT OF INTEREST STATEMENT

The authors declare no conflicts to declare.

## Data Availability

The data that support the findings of this study are available from the corresponding author upon reasonable request.
